# Supplementary material for: Challenges and experiences in linking community level reported out-of-pocket health expenditures to health provider recorded health expenditures: Experience from the iHOPE project in Northern Ghana
Source: PLoS One. 2021 Sep 7;16(9):e0256910. doi: 10.1371/journal.pone.0256910 (PMC8423291; doi:10.1371/journal.pone.0256910)
Supplement: S1 File — (DOCX) [file pone.0256910.s001.docx]

**Supplementary material 1**

**Health provider data template**

| **PROVIDER NAME:** | |  | **PROVIDER CODE:** |  |  | **PROVIDER TYPE:** |  |  |  |  |
| --- | --- | --- | --- | --- | --- | --- | --- | --- | --- | --- |
| **sn** | **Name of Client** | **Address/Location** | **Insured (Y/N)** | **Prescribed (Y/N)** | **Diagnosis** | **Drug name** | **Qua'ty** | **Unit cost (GHC)** | **Total Cost** | **Date** |
|  |  |  |  |  |  |  |  |  |  |  |
|  |  |  |  |  |  |  |  |  |  |  |
|  |  |  |  |  |  |  |  |  |  |  |
|  |  |  |  |  |  |  |  |  |  |  |
|  |  |  |  |  |  |  |  |  |  |  |
|  |  |  |  |  |  |  |  |  |  |  |
|  |  |  |  |  |  |  |  |  |  |  |
|  |  |  |  |  |  |  |  |  |  |  |
|  |  |  |  |  |  |  |  |  |  |  |
|  |  |  |  |  |  |  |  |  |  |  |
|  |  |  |  |  |  |  |  |  |  |  |
|  |  |  |  |  |  |  |  |  |  |  |
